# Prevalence and comorbidity of attention deficit hyperactivity disorder in Spain: study protocol for extending a systematic review with updated meta-analysis of observational studies

**DOI:** 10.1186/s13643-019-0967-y

**Published:** 2019-02-11

**Authors:** Ferrán Catalá-López, Manuel Ridao, Amparo Núñez-Beltrán, Ricard Gènova-Maleras, Adolfo Alonso-Arroyo, Rafael Aleixandre-Benavent, Miguel A. Catalá, Rafael Tabarés-Seisdedos

**Affiliations:** 1 Department of Health Planning and Economics, National School of Public Health, Institute of Health Carlos III, Madrid, Spain; 2 Department of Medicine, University of Valencia/INCLIVA Health Research Institute and CIBERSAM, Valencia, Spain; 3 Fundación Instituto de Investigación en Servicios de Salud, Valencia, Spain; 4 Instituto Aragonés de Ciencias de la Salud (IACS), Red de Investigación en Servicios de Salud en Enfermedades Crónicas (REDISSEC), Zaragoza, Spain; 5 Independent Clinical Psychologist and Researcher, Madrid, Spain; 6 Directorate General for Public Health, Madrid Regional Health Council, Madrid, Spain; 7 Department of History of Science and Documentation, University of Valencia, Valencia, Spain; 8 Information and Social and Health Research Unit (UISYS), University of Valencia and Spanish National Research Council (CSIC), Valencia, Spain; 9 Institute for Innovation and Knowledge Management (INGENIO)/Spanish National Research Council (CSIC) and Polytechnic University of Valencia (UPV), Valencia, Spain; 10 Faculty of Medicine, University of Valencia, Valencia, Spain

**Keywords:** Attention deficit hyperactivity disorder, Comorbidity, Epidemiological study, Meta-analysis, Prevalence, Spain, Systematic review

## Abstract

**Background:**

Attention deficit hyperactivity disorder (ADHD) is a childhood-onset disorder characterized by a persistent pattern of symptoms of developmentally inappropriate and impaired inattention and/or hyperactivity/impulsivity, with difficulties often continuing into adulthood. ADHD can come with other comorbid conditions. The aim of this study will be to quantify the prevalence and comorbidity of ADHD among children, adolescent, and adult population in Spain.

**Methods/design:**

We designed and registered a study protocol for an update and expansion of a systematic review and meta-analysis of pooled prevalence data. We will include cross-sectional observational studies reporting prevalence of ADHD in Spain and conducted in the general population, outpatient, and/or school settings. The primary outcome will be the prevalence of ADHD. Secondary outcomes will be the prevalence of any physical or mental comorbidity in association with ADHD. No limitations will be imposed on publication status, study conduct period, and language of dissemination. Comprehensive literature searches will be conducted in multiple electronic databases, including PubMed/MEDLINE, EMBASE, Scopus, Web of Science, PsycINFO, IME – Spanish Medical Index, and IBECS – Spanish Bibliographic Index of Health Sciences. We will also search Google Scholar, dissertation databases, and conference abstracts. Two team members will independently screen all citations, full-text articles, and abstract data. Potential conflicts will be resolved through discussion. The methodological quality (or risk of bias) of individual studies will be appraised using an appropriate tool. If feasible, we will conduct random effects meta-analysis. Prevalence estimates will be stratified according to gender, age, and geographical location. Additional analyses will be conducted to explore the potential sources of heterogeneity (e.g., methodological quality, sample size, diagnostic criteria).

**Discussion:**

This systematic review and meta-analysis of observational data will provide an updated synthesis of the prevalence and comorbidity of ADHD in Spain. This study will also examine factors that may explain potential variations in prevalence data. The findings of this study will be published in a peer-reviewed journal.

**Systematic review registration:**

PROSPERO CRD42018106082.

**Electronic supplementary material:**

The online version of this article (10.1186/s13643-019-0967-y) contains supplementary material, which is available to authorized users.

## Background

Attention deficit hyperactivity disorder (ADHD) is a childhood-onset disorder characterized by a persistent pattern of symptoms of developmentally inappropriate and impaired inattention and/or hyperactivity/impulsivity, with difficulties often continuing into adulthood [1–3]. ADHD is more common in boys than in girls [1, 4] and can often come with other conditions (the so-called comorbidity) [5–7]. Considerable debate exists surrounding the diagnosis [8–13] and treatment [14–17] of ADHD. Claims for the condition being overdiagnosed or underdiagnosed underscore the importance of rigorous assessment [1, 11]. Recent prevalence estimates suggest that ADHD affects about 3–7% of young people worldwide [18–23], producing considerable impact on health services and the community [18, 24, 25]. However, prevalence estimates of ADHD within and between countries often vary widely [19, 20, 26], and reports of increases in prevalence further fuel the controversy [19].

Systematic reviews of descriptive epidemiology are important for characterizing the amount and geographical distribution of health problems. Meta-analysis of epidemiological data (whether at a national, regional or global levels) can be useful to get more precise estimates of disease frequency, monitor trends and changes in disease burden over time, and establish a benchmark pooled prevalence but also to examine whether estimates have increased (or decreased) with publication of different study features. Previous systematic reviews and meta-analyses have traditionally assessed the worldwide prevalence of ADHD based on studies from a broad geographic distribution (e.g., North America, Europe, Asia) [19–22]. Very few meta-analyses exist in the biomedical literature reporting ADHD prevalence estimates at the country level [27–29].

In 2011, members of our review team conducted a meta-analysis of ADHD prevalence in Spain from 14 observational studies and more than 13,000 children and adolescent participants [29]. The main findings were published in 2012, suggesting a first pooled prevalence estimate of 6.8% (95% confidence interval: 4.9 to 8.8%) at the country level [29]. The 2011 review results drew attention in particular to ADHD among children and adolescents [30, 31], but did not include aspects such as comorbidity or adult population. In addition, overall findings were limited by clinical and methodological heterogeneity [29]. In recent years, several (new) epidemiological studies have been conducted in different geographical locations and population groups [32–34]. In addition, methods have advanced quickly; planning data extraction and analyses and understanding of the review process have become more sophisticated [35–41]. Therefore, we consider it timely to update and expand on our previous systematic review and meta-analysis [29] with much more detailed analysis of relevant data.

The objective of this study will be to quantify the prevalence and comorbidity of ADHD among children, adolescent, and adult population in Spain and to examine factors that may explain the variations in prevalence based on the latest evidence from descriptive epidemiology.

## Methods

### Protocol

This study protocol is part of an ongoing evidence synthesis project on the descriptive epidemiology and surveillance of neurodevelopmental disorders [42]. The present protocol has been registered within the PROSPERO database (registration number: CRD42018106082) and is being reported in accordance with the reporting guidance provided in the Preferred Reporting Items for Systematic Reviews and Meta-Analyses Protocols (PRISMA-P) statement [39, 40] (see checklist in Additional file 1).

### Information source and literature search

The primary source of literature will be a structured search of major electronic databases, including PubMed/MEDLINE, EMBASE, Scopus, Web of Science, and PsycINFO, but also national databases including IME – *Índice Médico Español* [Spanish Medical Index] and IBECS – *Índice Bibliográfico Español en Ciencias de la Salud* [Spanish Bibliographic Index of Health Sciences]). The secondary source of potentially relevant material will be a search of the gray or difficult to locate literature, including two dissertation databases (TESEO – *Base de datos de Tesis Doctorales* [Spanish Data Base of Doctoral Thesis Dissertations] and ProQuest Dissertations and Theses Database), Google Scholar, and conference abstracts from selected national or local symposia on mental health, neurology, and pediatrics. We will perform hand-searching of the reference lists of included studies, relevant reviews, national clinical practice guidelines, or other relevant documents (e.g., official documents and government reports). Content experts and authors who are prolific in the field will be contacted. The literature searches will be designed and conducted by the review team which includes two experienced health information specialists. Our main literature search will be peer-reviewed by a senior health information specialist using the Peer Review of Electronic Search Strategies (PRESS) checklist [41]. The search will include a broad range of terms and keywords related to ADHD, epidemiological studies, and the geographical area “Spain.” For the section of geographic area, the search will be based on a previously validated filter to minimize potential bias regarding the indexing of geographical items [43]. This filter is constructed around three complementary approaches: (1) the term “Spain” and its variants in various languages, (2) terms related mainly to region and province place names, and (3) acronyms for regional health services. A draft search strategy for PubMed/MEDLINE is provided in Additional file 2.

### Eligibility criteria

Studies will be selected according to the following criteria: participants, condition or outcome(s) of interest, study design, and context.

- Participants (population): We will include studies involving children, adolescents, and adult population (regardless of age or sex).
- Condition or outcome(s) of interest: The primary outcome will be the prevalence of ADHD indicating the number of people that have the disorder divided by the population number at a given point in time. This is often presented as a (prevalence) proportion. We will use author-reported definitions (according to accepted diagnostic criteria, such as the Diagnostic and Statistical Manual of Mental Disorders [DSM] or the International Classification of Diseases [ICD] criteria: ICD-9: 314.00, 314.01; ICD-10: F90). Secondary outcomes will be the prevalence of any comorbidity, indicating the existence of any distinct additional (physical or mental) condition in association with ADHD (e.g., according to main DSM-IV, DSM-V, ICD-9, or ICD-10 categories of diagnoses).
- Study design and context: Eligible studies will be observational studies (cross-sectional or health surveys) reporting prevalence data using validated or non-validated tools and conducted in a wide range of people in the Spanish general population, outpatient (including data from administrative databases and registries), and/or school settings. Cross-sectional studies will be the most appropriate study design to determine the prevalence of ADHD. Cross-sectional health surveys are typically used to estimate the point prevalence of common conditions of long duration. We will exclude studies in hospital/inpatient clinical settings because they are likely to be highly selected resulting in inaccurate estimations of the “true prevalence” of the disorder.

No limitations will be imposed on language, publication status (unpublished studies will be eligible for inclusion), and study conduct period.

### Screening and selection procedure

All articles identified from the literature search will be screened by two team members independently. First, titles and abstracts of articles returned from initial searches will be screened based on the eligibility criteria outlined above. Second, full texts will be examined in detail and screened for eligibility. Third, references of all considered articles will be hand-searched to identify any relevant report missed in the search strategy. Any disagreements will be resolved by discussion to meet a consensus, if necessary. A flow chart showing details of studies included and excluded at each stage of the study selection process will be provided [44].

### Data collection

A data extraction form will be designed and used to extract equivalent information from each study report. Information of interest will include the following:

- Study characteristics: study design, year of publication, journal, year (or period) of study conduct, sample size, setting (community, school or outpatient), geographical location of study conduct: North (Galicia, Asturias, Cantabria, Aragon, Basque Country, Navarre, La Rioja), Mediterranean (Balearics, Catalonia, Valencia), Centre (Castile-La Mancha, Castile-León, Madrid, Extremadura), and South-East (Andalusia, Murcia and Canary Islands); and other fields to capture data relevant to the assessment of study methodological quality (see risk of bias assessment subsection).
- Participant characteristics: population sampled, age (e.g., mean with standard deviation, range) and gender (e.g., percentage of female participants).
- Outcome results: definitions and measures used to make diagnosis (e.g., symptom only checklists, reports of diagnosis, interviews and examination) and whether the diagnosis met the full DSM/ICD criteria for each edition (e.g., DSM-III, DSM-IV, DSM-V, ICD-9, ICD-10, ICD-11, other), informants or people reporting symptoms for a diagnostic evaluation (e.g., child, clinician, parent, teacher and/or a combination rule), prevalence estimates (e.g., number of subjects with the disorder, proportion and 95% confidence interval), and any prevalence estimates stratified by age, gender, severity (impairment criterion for DSM-IV), or location. The most conservative diagnosis will be used in those studies reporting more than one prevalence estimate. If outcome results (e.g., proportion and 95% confidence interval) are not directly provided and it is feasible, we will calculate them from the number of cases and sample size provided in each single study.

Data extraction forms will be piloted initially on a small number of included studies. Subsequently, each of the included studies will be abstracted by two team members, independently, and potential conflicts will be resolved through discussion. Authors of primary publications (e.g., corresponding authors) will be contacted by email for data clarifications or missing outcome data, as necessary. First, authors will be sent an email requesting their missing outcome data or data clarifications. Second, we will send three email reminders at 2-, 6-, and 10-week intervals after the initial email. In cases where the identified studies do not report authors’ email addresses or include non-working email addresses, we will search authors’ publications, PubMed, and profiles that are publicly available (e.g., ORCID, ResearchGate, and Google Scholar), to find contact information.

### Risk of bias in individual studies

The risk of bias in individual studies will be evaluated using a methodological quality critical appraisal checklist proposed by the Joanna Briggs Institute (JBI) systematic review methods manual [37, 38]. This tool for observational studies reporting prevalence data considers the following: sample representativeness, recruitment appropriateness, sample size, description of subjects and setting, coverage of data analysis, ascertainment and measurement of the condition, thoroughness of reporting statistical analysis, and identification and accountability of potential confounding factors/subgroups (see Additional file 3). Stars or points will be awarded for each quality item, and the highest quality studies will be awarded up to ten stars. Studies will be judged to be at low risk of bias (≥ 7 points), moderate risk of bias (4–6 points), or high risk of bias (< 4 points) [42]. The risk of bias for each individual study will be independently assessed by two reviewers. Discrepant scores will be resolved by discussion and consensus. We will provide a narrative summary of the risk of bias of the included studies, which will be supported by a table showing the results of the critical appraisal.

### Methods for evidence synthesis

The data from each paper (e.g., study characteristics, context, participants, outcomes and findings) will be used to build evidence tables of an overall description of included studies. Crude prevalence estimates (number of cases/sample size) will be presented along with 95% confidence intervals. If feasible and appropriate, prevalence data points from primary observational studies will be used to perform random effects meta-analyses. Since heterogeneity is expected a priori, we will estimate the pooled prevalence and its 95% confidence interval using the random effects model with logit transformation and back transformation [42]. The random effects model assumes the study prevalence estimates follow a normal distribution, considering both within-study and between-study variation. Forest plots will be used to visualize pooled estimates and the extent of heterogeneity among studies.

We will quantify statistical heterogeneity by estimating the variance between studies using *I*^*2*^ statistic [45]. The *I*^*2*^ statistic is the proportion of variation in prevalence estimates that is due to genuine variation in prevalence rather than sampling (random) error. *I*^*2*^ statistic ranges between 0 and 100% (with values of 0–25% and 75–100% taken to indicate low and considerable heterogeneity, respectively). We will also report Tau^2^ [46] and Cochran Q test [47] with a *P* value of < 0.05 considered statistically significant (heterogeneity).

### Additional analyses

If sufficient studies are identified and data points are available, potential sources of heterogeneity will be investigated further by subgroup or meta-regression analyses [48] according to baseline characteristics and methodological covariates [1–4, 19, 29, 42]. We plan to conduct analyses by gender (male vs female), age (e.g., children vs adolescent vs adult, mid-point of age range as continuous variable), geographical location (e.g., North, Mediterranean, Centre, and South-East), setting (e.g., community/school vs outpatient), sample size (e.g., < 500, 500–1500, or > 1500 participants), decade of publication (e.g., 1990, 2000, or 2010), study quality (e.g., low/moderate vs high risk of bias), diagnostic system (e.g., DSM vs ICD criteria), and most recent diagnostic criteria (e.g., “DSM-IV/V or ICD-10/11” vs “Not DSM-IV/V or ICD-10/11”). In addition, we will explore prevalence trends with gender variations (in terms of the female-to-male prevalence ratios) and over time (with the year of publication as the explanatory variable) using random effects meta-regression models [29].

### Meta-bias

Small study effects (or “publication bias” across studies) will be assessed by inspection of the funnel plots for asymmetry and with Egger’s test [49] and Begg’s test [50], with the results considered to indicate potential small study effects when *P* values are < 0.10.

### Software considerations

All analyses will be conducted in Stata version 15 (StataCorp LP, College Station, Texas, USA) [51, 52].

### Patient and public involvement

We will evaluate whether the epidemiological studies included in the systematic review had any patient and public involvement [53, 54].

No patients and/or public were involved in setting the research question for this study nor were they involved in developing plans for design (or implementation) of this protocol. No patients and/or public will be asked to advise on interpretation or writing up of results.

### Ethics, dissemination, and research integrity

No ethical approval is required for the performance of this study. The proposed systematic review and meta-analysis will be reported in accordance with the reporting guidance provided in the Preferred Reporting Items for Systematic Reviews and Meta-analyses (PRISMA) statement [44] and the Meta-analysis Of Observational Studies in Epidemiology (MOOSE) reporting guideline [55]. Any amendments made to this protocol when conducting the study will be outlined and reported in the final manuscript. Results will be disseminated through conference presentations and publication in a peer-reviewed journal. All data underlying the findings reported in the final manuscript will be deposited in a cross-disciplinary public repository—such as the Open Science Framework (https://osf.io/) or Zenodo (https://zenodo.org/).

## Discussion

Up-to-date epidemiological evidence about levels and trends in country-specific morbidity (such as prevalence) is essential input into national and subnational health policy and planning debates. In fact, the availability of national prevalence estimates of ADHD provides opportunities to undertake further systematic assessments of the burden of disease [18, 23, 25].

In this paper, we have presented a study protocol for extending a systematic review with updated meta-analysis of the prevalence and comorbidity of ADHD in Spain. This protocol updates and expands methods for a new systematic review that will supersede previous meta-analyses of observational studies on this topic [29]. The improved approaches to the methods, analysis, and refinements in epidemiological data (revisions and updates, exploration of the extent of bias, heterogeneity, and potential variations in prevalence data), as well as the widening of scope by age (e.g., children, adolescents and adults), causes of comorbidity (e.g., physical or mental), and time considering new data (e.g., studies from 1980 to 2020) are all relevant to this study.

A key challenge is that based on knowledge from previous systematic reviews on mental health [19–24, 56, 57], we anticipate identifying epidemiological studies with different features, populations, and contexts and with a variable quality of reporting methods and results. This study will identify knowledge gaps to be filled by new research. On this regard, implications for future epidemiological studies will be discussed in the final manuscript.

## Additional files

Additional file 1: PRISMA-P Checklist. (DOCX 33 kb)

Additional file 2: Key terms for PubMed/MEDLINE search. (DOCX 29 kb)

Additional file 3: Methodological Quality Checklist for Prevalence data. (DOCX 29 kb)

## Abbreviations

ADHD: Attention deficit hyperactivity disorder
DSM: Diagnostic and Statistical Manual of Mental Disorders
ICD: International Classification of Diseases
MOOSE: Meta-analysis Of Observational Studies in Epidemiology
PRISMA: Preferred Reporting Items for Systematic Reviews and Meta-Analyses
PRISMA-P: Preferred Reporting Items for Systematic Reviews and Meta-Analyses extension for Protocols
