# Supplementary material for: Prevalence and comorbidity of attention deficit hyperactivity disorder in Spain: study protocol for extending a systematic review with updated meta-analysis of observational studies
Source: Syst Rev. 2019 Feb 11;8:49. doi: 10.1186/s13643-019-0967-y (PMC6371515; doi:10.1186/s13643-019-0967-y)
Supplement: Supplementary file 3 — Methodological Quality Checklist for Prevalence data. (DOCX 29 kb) [file 13643_2019_967_MOESM3_ESM.docx]

**Additional file 3: Methodological Quality Checklist for Prevalence data (.docx)**

Note: This scale has been adapted from the JBI Critical Appraisal Checklist for Studies Reporting Prevalence Data. The individual components listed below are summed to generate a total Methodological Quality score for each study. Total scores range from 0 to 10. For the total score grouping, studies were judged to be of low risk of bias (≥7 points), moderate risk of bias (4-6 points) and high risk of bias (<4 points).

1) **Was the sample representative of the target population?**

a) Yes**🟑**

b) No

c) Unclear/no description

d) Not applicable

2) **Were study participants recruited in an appropriate way?**

a) Ye (e.g. sampling frame, random selection)**🟑**

b) No

c) Unclear/no description

d) Not applicable

3) **Was the sample size adequate?**

a) Yes**🟑**

b) No

c) Unclear/no description

d) Not applicable

4) **Were the study subjects and the setting described in detail?**

a) Yes**🟑**

b) No

c) Unclear/no description

d) Not applicable

5) **Was the data analysis conducted with sufficient coverage of the identified sample?**

a) Yes (e.g. non-response bias minimal)**🟑**

b) No

c) Unclear/no description

d) Not applicable

6) **Were objective, standard criteria used for measurement of the condition?**

a) Yes**🟑**

b) No

c) Unclear/no description

d) Not applicable

7) **Was the condition measured reliably?**

a) Yes**🟑**

b) No

c) Unclear/no description

d) Not applicable

8) **Was there an appropriate reporting of statistical analysis?**

a) Yes**🟑**

b) No

c) Unclear/no description

d) Not applicable

9) **Are all important confounding factors, subgroups, or potential differences identified and accounted for?**

a) Yes**🟑**

b) No

c) Unclear/no description

d) Not applicable

10) **Were subpopulations identified using objective criteria?**

a) Yes**🟑**

b) No

c) Unclear/no description

d) Not applicable
